# Associations Between Stress Exposure and Emotional Health: The Moderating Role of Discrimination

**DOI:** 10.1093/geroni/igab046.2148

**Published:** 2021-12-17

**Authors:** Daisy Zavala, Elizabeth Muñoz, Martin Sliwinski, Stacey Scott

**Affiliations:** 1 Stony Brook University, Ridgewood, New York, United States; 2 University of Texas at Austin, University Of Texas At Austin, Texas, United States; 3 The Pennsylvania State University, University Park, Pennsylvania, United States; 4 Stony Brook University, Stony Brook, New York, United States

## Abstract

The Weathering Hypothesis states BIPOC face more stressors, by which over a lifetime they are subjected to the negative consequences of stress (e.g., poorer emotional health). Using ecological momentary assessments, we examined whether subtle discrimination moderated the within-person stressor slope on positive and negative affect. We predicted emotional wellbeing would be worse at stressor moments, and those with greater discrimination experience would be more impacted by stressors. Participants were 334 diverse adults (25-65 years, Mage = 47, 63% Female) from Bronx, New York. Positive affect decreased and negative affect increased significantly at stressor moments (p<.0001). Unexpectedly, subtle discrimination was not a significant moderator for the within-person stressor slope on positive affect and negative affect. Unlike the predictions of the Weathering Hypothesis, these results show that prior discrimination experiences may not exacerbate responses to stressors and entail additional risk in daily life.

